# Doctor-patient interactions in the age of AI: navigating innovation and expertise

**DOI:** 10.3389/fmed.2023.1241508

**Published:** 2023-08-30

**Authors:** Brett N. Hryciw, Zanna Fortin, Jamie Ghossein, Kwadwo Kyeremanteng

**Affiliations:** ^1^Department of Medicine, Division of Critical Care, University of Ottawa, Ottawa, ON, Canada; ^2^Gemeinschaftspraxis im Bayerwald, Bavaria, Germany; ^3^Department of Medicine, University of Ottawa, Ottawa, ON, Canada; ^4^Clinical Epidemiology, Ottawa Hospital Research Institute, University of Ottawa, Ottawa, ON, Canada; ^5^Institute du Savoir Montfort, Ottawa, ON, Canada

**Keywords:** artificial intelligence, healthcare, decision-making, patient care, ethics, medical practice

## Abstract

The integration of artificial intelligence (AI) in healthcare has the capacity to transform medical practice. Despite its revolutionary potential, the influence of AI may affect the physician-patient interaction and presents ethical challenges that will need to be carefully considered. This article discusses how patients may interact with this technology, considers how emerging technologies may alter the dynamics of the physician-patient relationship, and reviews some of the limitations that continue to exist. We identify potential challenges that may arise with the integration of AI into medical settings and propose solutions to help mitigate these issues.

## Introduction

The adoption of artificial intelligence (AI) in healthcare has the potential to revolutionize medical practice, improving diagnostics, treatment planning, and overall patient care (1). However, the integration of AI into clinical settings also presents new challenges for doctor-patient interactions, as well as ethical concerns that must be carefully considered. In this article, we will explore the complexities of patients and families introducing AI-generated medical opinions into doctor-patient relationships and discuss strategies for effectively navigating these challenges.

### Patients as technology consumers

With the development of the internet, patients have been increasingly empowered to become informed about their health, allowing them to access a wealth of medical information from various sources. As a result, patients can take an increasingly active role in their healthcare decision-making (2). This has both positive and negative implications for doctor-patient relationships. For instance, when patients bring internet-generated opinions to their medical appointments, it can promote informed discussions and better decision-making. On the other hand, patients may develop rigid beliefs about optimal medical management based on internet advice, which may not align with the doctor’s professional opinion, potentially straining the therapeutic alliance (3).

### Addressing the shift in dynamics

Similarly, as AI-generated medical opinions become more accessible and reliable, patients may turn to AI software to provide advice regarding their healthcare. Consequently, the rise of AI-generated medical opinions will likely lead to a further shift in dynamics between physicians, who historically held all the knowledge and expertise, and patients or family members, who can now access AI-generated opinions with increasing sophistication and accuracy through large language model (LLM) chatbots such as OpenAI’s GPT-4 or Google’s Bard. A medicine specific consumer technology, Glass AI, is a GPT-4 based technology where users present a clinical scenario and subsequently a differential diagnosis or clinical management plan is generated. As these technologies become more mainstream, it seems likely that patients will arrive to clinical encounters with specific expectations for next steps in their care. The advantage of AI lies in its ability to process large volumes of data and identify patterns that may not be readily apparent to human clinicians, which has revolutionary potential in the age of AI innovation (4, 5). Already these technologies have been applied to a range of clinical scenarios and, in select circumstances, may be able to recognize biological signatures in patient data that is beyond human interpretation. In fact, an automated deep learning model of retinal fundus photographs from a UK database was able to reliably predict a patient’s reported sex which is beyond human capabilities (6).

While empowerment of patients and families to participate in health care decision is undeniably important, those who choose to seek AI-generated medical opinions could strain doctor-patient relationships if the physician feels threatened or if families do not accept the current limitations of these tools and believe that the AI-generated opinions are superior. This possibility necessitates a more collaborative approach in doctor-patient relationships, emphasizing partnership and shared decision-making with an openness to discussing AI-generated opinions. Physicians should be encouraged to embrace this shift and actively engage patients and families as partners in the decision-making process, acknowledging the value of AI-generated insights while maintaining their unique role as human experts (7).

### Identifying ethical concerns

Amidst the evolving domain of AI ethics, its implications in medicine raise concerns of informed consent, training biases, and transparency among others. Firstly, informed consent is a core component of medical ethics but has the potential to be compromised by providing misinformation. AI algorithms are not infallible and can produce false or misleading information, known as AI hallucinations. These errors can arise from biases in the training data or limitations in the AI’s understanding of complex medical scenarios (8, 9). Overreliance on AI-generated opinions by patients may in fact lead to suboptimal healthcare decisions and outcomes when the uniqueness of individual patients, the broader clinical context, and the expertise of human clinicians are not appropriately considered. Further, with the potential for unrecognized AI hallucinations, knowledge provided to the patient and families has the potential to bias and misinform patients, in turn clouding judgement. This is particularly relevant as patients receive and place increasing value on AI-generated advice without understanding its limitations. This may in fact compromise patient autonomy and lead to ill-informed decision-making (7, 10). Additionally, LLMs are limited by their training data set. Inherent biases can arise when AI is trained on non-representative patient data, potentially leading to less accurate predictions for underrepresented populations or diseases (8, 11). Unfortunately, underrepresentation biases often further disadvantage marginalized populations. Consequently, physicians may be obliged to educate patients while relying on their expertise and judgment to interpret AI advice in the context of the individual patient’s condition and needs, helping to mitigate potential biases. Transparency is a cornerstone of the evolving physician-patient relationship in the era of AI-driven healthcare. As AI systems can sometimes be perceived as “black boxes” with their complex decision-making processes, physicians must highlight that while AI can provide useful information, it may not yet consider all relevant factors or nuances of a patient’s unique circumstances that are considered by a human physician. Lastly, the question of who bears the responsibility when AI-based decisions lead to poor patient choices or adverse patient outcomes remains an ongoing debate. Clear guidelines on responsibility attribution and informed consent procedures are needed to address this issue.

### Involving patients in decision-making processes

Patient involvement in the decision-making process is paramount for promoting responsible AI integration in healthcare. By engaging patients with AI-generated insights, physicians can ensure that these are considered alongside human expertise and experience, as well as the patient’s preferences and unique circumstances (12). Patient-centered care models, which focus on active collaboration between patients, families, and healthcare providers, can help achieve this goal (13). By fostering a patient-centered approach, healthcare providers can maintain the human element of care while leveraging the benefits of AI-generated medical opinions.

Healthcare providers must also be educated about AI’s capabilities and limitations, enabling them to effectively explain AI-generated opinions to their patients (14). This can be achieved through targeted training programs and patient education initiatives, promoting ethical AI adoption and informed decision-making. By enhancing patient awareness of AI’s capabilities and limitations, healthcare providers can help to ensure that patients make well-informed decisions based on a combination of human expertise and AI-generated insights (10). Nevertheless, when an AI-generated opinion that resonates with patients or families differs from a doctor’s recommendation, this discrepancy may deter patients and family members from accepting the medical opinion. Clinicians must be prepared to explain their reasoning and engage in open conversations with patients to address potential concerns. Transparent communication is essential in maintaining trust and fostering collaborative decision-making in the age of AI.

### Future directions

It seems increasingly inevitable that AI, much like the internet previously, will permeate many aspects of society. Almost certainly, patients be among AI consumers who will turn to these technologies to illicit medical advice when access to healthcare is not readily available. As AI tools become increasingly reliable, validated consumer tools should be trained and validated using data that encompasses the local diversity of the patient population they are meant to service. This approach could reduce potential underrepresentation biases and disparities in healthcare outcomes and enhance the tool’s relevance and effectiveness. One of the most compelling advantages of AI is its potential to alleviate limitations in healthcare access. For example, AI tools may eventually be capable of triaging patient concerns, identifying those requiring immediate attention and those suitable for virtual consultations. This would not only enhance resource allocation but also extend the reach of healthcare to underserved populations.

Concurrently, the importance of AI training programs for medical professionals cannot be overstated. As we transition into an era of AI-augmented healthcare, it’s essential that our doctors, nurses, and other healthcare workers are equipped with the necessary skills to navigate this new landscape. They need to understand how to integrate AI-generated advice into their practice and communicate these insights effectively to patients. This training will not only augment their ability to provide care but also bolster their confidence as they navigate this new frontier in medicine. Patient education and engagement are equally vital. Patients, now more than ever, are active participants in their healthcare journey. As such, they must be equipped with a basic understanding of AI’s strengths and limitations. Educational resources or initiatives could help patients make sense of AI-generated insights, promoting informed discussions and decision-making while promoting trust in their healthcare providers.

Additionally, as we grapple with the ethical and logistical aspects of AI deployment, longitudinal studies can offer much-needed insight into AI’s real-world impact over time. Concurrently, a cost–benefit analysis is crucial. While AI’s immense potential cannot be understated, the cost associated with integrating AI into healthcare systems must be justifiable.

## Conclusion

The integration of AI into healthcare is inevitable and offers many benefits for patient care. However, it is crucial to address potential challenges in doctor-patient interactions and maintain trust in the face of AI-generated medical opinions. By fostering open communication, recognizing AI’s limitations, and valuing human expertise, clinicians can successfully navigate the evolving landscape of healthcare and ensure the best possible care for their patients. The education for healthcare providers and involving patients in decision-making processes are essential strategies for the responsible integration of AI in healthcare. As we move forward with integrating AI into healthcare, it’s paramount that we do so with a thoughtful and comprehensive approach. Ensuring effective regulation, standardization, and education will pave the way for a healthcare landscape where AI is not just a tool for doctors, but an ally for patients as well.

## Data availability statement

The original contributions presented in the study are included in the article/supplementary material, further inquiries can be directed to the corresponding author.

## Author contributions

KK and BH contributed to the conceptualization of the paper. AI-generated content from structured and refined input data using GPT-4 was overseen by BH. BH, ZF, and JG monitored content generation iteratively and were responsible for editing, revising and validating all AI-generated content to ensure reliability and accuracy. KK was responsible for overseeing project completion. All authors contributed to the article and approved the submitted version.

## Conflict of interest

The authors declare that the research was conducted in the absence of any commercial or financial relationships that could be construed as a potential conflict of interest.

## Publisher’s note

All claims expressed in this article are solely those of the authors and do not necessarily represent those of their affiliated organizations, or those of the publisher, the editors and the reviewers. Any product that may be evaluated in this article, or claim that may be made by its manufacturer, is not guaranteed or endorsed by the publisher.
